# IS THERE A RIGHT TIME TO RETIRE? AFFECTIVE DIS/ENGAGEMENT FROM WORK IN A LONGITUDINAL QUALITATIVE STUDY FROM GERMANY

**DOI:** 10.1093/geroni/igz038.078

**Published:** 2019-11-08

**Authors:** Anna Wanka

**Affiliations:** Goethe University Frankfurt am Main, Frankfurt am Main, Germany

## Abstract

Recent retirement research has argued that the once predictable pattern associated with retiring is becoming increasingly differentiated by the age at which it occurs, if it is gradual of abrupt, voluntary or involuntary, etc. (Moffatt & Heaven 2017). Even though research suggests that retirement legislation and statutory retirement ages influence the subjective perception of a ‘right time to retire’ (cf. Jansen, 2018), many people don’t feel ready to retire when they should and hence retire involuntarily (Steiber & Kohli, 2017). This paper focuses on the dis/engagement processes that lead to feeling ‘ready’ to retire at a certain time, or not. Drawing on data from a qualitative longitudinal study in Germany, results suggest (1) that readiness to retire is a continuum, rather than a binary, which is influenced by a variety of actors, and (2) that it is and a process that changes multiple times in the retirement transition.

